# Regulatory Mechanism of Trap Formation in the Nematode-Trapping Fungi

**DOI:** 10.3390/jof8040406

**Published:** 2022-04-16

**Authors:** Mei-Chen Zhu, Xue-Mei Li, Na Zhao, Le Yang, Ke-Qin Zhang, Jin-Kui Yang

**Affiliations:** State Key Laboratory for Conservation and Utilization of Bio-Resources, Key Laboratory for Microbial Resources of the Ministry of Education, School of Life Sciences, Yunnan University, Kunming 650091, China; zmc201789@163.com (M.-C.Z.); xmli@mail.ynu.edu.cn (X.-M.L.); zn0314@mail.ynu.edu.cn (N.Z.); yangle@mail.ynu.edu.cn (L.Y.); kqzhang1@ynu.edu.cn (K.-Q.Z.)

**Keywords:** nematode-trapping fungi, trap formation, signaling pathways, signal molecules, phenotype analysis, regulation mechanism, pathway collaboration

## Abstract

Nematode-trapping (NT) fungi play a significant role in the biological control of plant- parasitic nematodes. NT fungi, as a predator, can differentiate into specialized structures called “traps” to capture, kill, and consume nematodes at a nutrient-deprived condition. Therefore, trap formation is also an important indicator that NT fungi transition from a saprophytic to a predacious lifestyle. With the development of gene knockout and multiple omics such as genomics, transcriptomics, and metabolomics, increasing studies have tried to investigate the regulation mechanism of trap formation in NT fungi. This review summarizes the potential regulatory mechanism of trap formation in NT fungi based on the latest findings in this field. Signaling pathways have been confirmed to play an especially vital role in trap formation based on phenotypes of various mutants and multi-omics analysis, and the involvement of small molecule compounds, woronin body, peroxisome, autophagy, and pH-sensing receptors in the formation of traps are also discussed. In addition, we also highlight the research focus for elucidating the mechanism underlying trap formation of NT fungi in the future.

## 1. Introduction

Plant-parasitic nematodes (PPNs) can cause direct damage to their host or act as virus vectors [1]. There are more than 4100 species of PPNs that have an impact on global agriculture and horticulture, causing an estimated annual loss of USD 173 billion [2,3]. Nematode-trapping (NT) fungi are potential biocontrol resources, which have the advantages of low toxicity, high efficiency, and environmental friendliness, so they have gradually become favored by people in recent years [3]. NT fungi can develop specialized structures called “traps”, an important indicator of the transition from saprophytic to predatory lifestyles, including constricting rings, adhesive networks, adhesive columns, and adhesive knobs, to capture, kill, and consume nematodes at a nutrient-deprived condition [4,5]. Therefore, trap formation is a crucial step in the lifestyle of NT fungi and indispensable for nematode predation. The factors involved in trap formation were variety, including multiple signal transduction pathways [6]; small molecular compounds [7,8,9,10,11,12]; intercellular communication [13]; adhesive protein [5]; nitrate assimilation [14]; woronin body [15]; peroxisome [16]; autophagy [17,18,19,20]; and pH-sensing receptors [21], according to the comprehensive research of genomics, transcriptomics, proteomics, metabolomics, and reverse genetics. Here, we review the recent progress in the regulatory mechanism of trap formation in the NT fungi based on phenotypes of various mutants and multi-omics analysis. We especially focus on the latest studies in signal transduction pathways and small molecular compounds involved in trap formation. Elucidating the molecular mechanism of trap formation will not only provide a theoretical basis for the improvement of engineering biocontrol fungi, but contribute to understanding the adaptive evolution and lifestyle transition of NT fungi.

## 2. Multi-Omics Analysis Promotes Research on Trap Formation of NT Fungi

Omics not only provides a macroscopic direction for the mechanism of trap formation but also provides specific targets. Comparative genomics studies have shown that many species-specific genes have expanded in the evolutionary process, and these genes may be related to the function specialization of NT fungi [5,6,22,23]. *Arthrobotrys oligospora* (teleomorph *Orbilia auricolor*), one of the typical NT fungi, was the first NT fungus whose genome and proteome were sequenced [6]. Comparative analysis showed that *A. oligospora* genome contains numerous pathogenicity-related genes, and a total of 398 homologous genes related to the pathogenicity of other fungi have been identified [6], while there were fewer lectin genes involved in fungus–nematode recognition in the *Drechslerella stenobrocha* genome [22]. This suggested that a different mechanism of trap formation may exist in various NT fungi. In addition, studies combined genome, proteome, and real-time PCR (RT-PCR) analyses to reveal that multiple signal transduction pathways have an integral part in trap formation [6]. Transcriptome sequencing and RT-PCR analysis showed that a large number of genes were significantly upregulated during the infection process of NT fungi, including genes involved in translation, amino acid metabolism, carbohydrate metabolism, cell wall and membrane biogenesis [6], secreted proteins [23], adhesion proteins [5], and the protein kinase C signal transduction pathway [22]. Comparative genomic analysis in the four representative trapping devices of NT fungi suggested that the simplification of the capture device was accompanied by the expansion of adhesion genes and the increase in adhesiveness on trap surfaces [5]. In conclusion, the above studies show that omics technologies have clarified the general direction for research on the regulation mechanism of traps formation. At the same time, the assembly of genomes in different fungi makes it possible to study the interaction between the NT fungi and nematodes at the molecular level. For instance, according to the genome sequence and annotation of *Duddingtonia flagrans*, a fluorescent protein system with native promoter was established, and a secretion protein PEFB was identified which was involved in the processes of infection against *Caenorhabditis elegans* [13]. Recently, nine species of NT fungi have been sequenced, such as adhesive-network-producing fungi *A. oligospora* and *D. flagrans*, adhesive-knob-producing fungi *Monacrosporium haptotylum* and *Dactylellina entomopaga*, constricting-ring-producing fungi *D. stenobrocha* and *Drechslerella brochopaga*, adhesive-columns-producing fungus *Dactylellina cionopagum*, and no trap producing fungus *Dactylella cylindrospora* (Table 1); these genomic information may help to elucidate the mechanism of trap formation of NT fungi.

## 3. Overview of Signaling Pathways Involved in Trap Formation

### 3.1. G-Protein Signaling Pathway Involved in Trap Formation

G-protein signaling is the most conserved signal transduction pathway in fungi, composed of heterotrimeric G-proteins (G-proteins), G-protein-coupled receptors (GPCRs), and regulators of G-protein signaling (RGSs), which plays a vital role in sensing the changes in various physical and chemical stimuli in the environment [25]. G-proteins have three subunits: Gα, Gβ, and Gγ. The latest research identified a single G-protein β-subunit gene (*gpb1*) in *A. oligospora*, and phenotypic analyses demonstrated that Δ*gpb1* mutants were strongly defective in the response to *C. elegans* and ascarosides, with the formation of few traps [24]. This means that GPCRs might be the receptor of ascarosides. In addition, G-protein signaling pathways are negatively regulated by RGSs. Recently, seven putative *rgs* genes were knocked out, and multi-phenotypic analyses shown that these genes affect the pathogenicity of *A. oligospora* to varying degrees. In particular, the Δ*flbA* deletion strains lost the ability to produce traps after being induced with nematodes [26]. In addition, resistance to inhibitors of cholinesterase 8 (RIC8), a conservative guanine nucleotide exchange factor, which is involved in the regulation of G-protein signaling in filamentous fungi. Recently, an orthologous RIC8 was characterized in *A. oligospora*, the Δ*ric8* deletion mutants lost the ability to produce traps essential for nematode predation, accompanied by a marked reduction in cyclic adenosine monophosphate (cAMP) level. Further assay revealed that RIC8 interacted with G-protein subunit Gα1 and is involved in nematode predation through control of cell cycle, organelle, and secondary metabolism [27].

The superfamily of small GTPases comprises signal transducers that regulate multiple cellular functions. RAS, RHO/RAC, RAB, ARF, and RAN are conserved groups of the small GTPases family that cycle between GTP-bound (active) and GDP-bound (inactive) conformations as a switch in signal transduction. Recently, two RAB GTPases were identified in *A. oligospora*: The Δ*rab-7A* deletion strains lost the ability to produce conidia and traps; however, the Δ*rab-2* mutants only slightly affected the conidiation but did not affect the trap formation [28]. In another research, three RAS GTPases were characterized by gene disruption and multi-omics analysis, the traps number of Δ*ras2* and Δ*rheb* deletion strains were significantly decreased, but Δ*ras3* mutants had no significant changes compared with the wild-type (WT) strain [29]. Our latest research demonstrated that three RHO GTPases (RHO2, RAC, and CDC42) played an important role in trap formation and lifestyle transition of *A. oligospora*. The *rac* was significantly upregulated, and alternative splicing events occurred in *rac* and *rho2* during the trap formation and infection process [30]. These studies indicated that the small GTPases have pleiotropic functions in the growth and development of *A. oligospora*; specifically, they play a very important role in trap formation and pathogenicity. Moreover, GTPase activating proteins (GAPs) are a family of proteins that induce the hydrolysis of GTP bound to small GTPases [31]. Recently, an ARF-GAP GLO3 was identified in *A. oligospora*. Trap formation was delayed; no nematodes were captured at nematode induction for 12 h in the Δ*glo3* mutants; and captured nematodes were significantly reduced at 24, 36, and 48 h compared with the WT strain [32]. Therefore, G-proteins and small GTPases play a pleiotropic role in the growth, development, trap formation, and pathogenicity in *A. oligospora* and other NT fungi.

### 3.2. Mitogen-Activated Protein Kinase (MAPK) Signaling Pathway Is Essential for Trap Formation

Accumulating evidences implicate that G-proteins can mediate signal transfer to MAPK-signaling cascade in filamentous fungi, which plays a significant role in pathogenicity [25]. Many NT fungi require very specific abiotic and biotic stimuli to form traps, the ability to sense and respond to environmental signals is essential during the trap formation and nematode predation [33]. MAPK signaling cascades are critical for pathogenic fungi to detect surrounding organisms in the environment [4,34]. There are three major MAPK cascades that have been well-studied in yeasts and filamentous fungi, including SLT2/MPK1 (cell wall integrity pathway), FUS3/KSS1 (pheromone-response and filamentous growth pathway), and HOG1 (hyperosmolarity pathway) [35,36]. Recent research showed that *slt2* was required for trap formation in two nematode-trapping fungi *A. oligospora* (strain ATCC24927) and *M. haptotylum* [34]. However, another study demonstrated that *slt2* is not essential for trap development in *A. oligospora* (strain TWF154), and its mutant was still able to develop traps after three days of nematode exposure [4]. Meanwhile, BCK1 and MKK1, two proteins of function upstream of SLT2, were mutants unable to produce spores and mycelial traps [37]. Moreover, the trap formation and predation efficiency were reduced in Δ*hog1* and Δ*msb2* mutants, and those mutants were highly sensitive to high osmolarity [38]. Moreover, SSK1, as an upstream regulatory protein of HOG1 signaling pathway, played a negative regulatory role in trap formation, as manifested by significantly increased trap formation and predation efficiency in Δ*ssk1* mutants [39]. In addition, deletion of *ste7* and *fus3* led to complete abolishment of conidiation and traps in mutants. In addition, STE12, a conserved transcription factor acting downstream of the pheromone-response pathway, induced disruption to defects in the response to nematode pheromone in *A. oligospora* [4]. Similarly, in *Drechslerella dactyloides*, the *Ddaste12* deletion strain cannot form inflationary contraction ring, which make it unable to catch nematodes [40]. In addition, inducer of meiosis 2 (IME2), a non-classical MAPK-pathway molecule, was associated with mycelial growth and development, conidiation, osmolarity, and pathogenicity in *A. oligospora*. The Δ*ime2* mutant cannot form mature traps containing multi-hyphal loop, and the electron-dense bodies in trap cells were less than the WT strain [41]. Together, these observations demonstrate that MAPK cascades are essential for trap formation of NT fungi.

### 3.3. cAMP-Dependent Protein Kinase A (cAMP/PKA) Signaling Pathway Is Indispensable for Trap Formation

The pathogenic process is related to infection-related formation and development in many pathogenic fungi, and the cAMP/PKA pathway plays an essential role in fungal pathogenesis [25]. In our latest research, the deletion of adenylate cyclase led to the abolishment of trap formation, and the number of traps in PKA subunits mutants were reduced in varying degrees (unpublished). In addition, the stunted protein STUA functions as the downstream of cAMP/PKA signaling pathway. The sporulation capacity of Δ*stuA* mutants were reduced 96%, and the ability to produce mycelial traps was lost [42]. Meanwhile, the transcriptional levels of several upstream genes of cAMP/PKA pathway were significantly reduced in Δ*stuA* mutants, such as *gpr*, *gα*, *ras2*, *acyA*, *pkaC1,* and *pkaC2*, and some downstream genes were also significantly downregulated, including *nsdc*, *cdc28*, and *cdc6* [42]. Similarly, the cAMP levels in Δ*ras2* and Δ*rheb* mutants were significantly lower than in the WT strain, and the downstream genes of the cAMP/PKA were significantly downregulated [29]. The cAMP level of the Δ*ric8* mutant was reduced to 3.0%–11.3% compared to the WT strain [27]. These findings indicate that the cAMP signaling pathway is indispensable for trap formation in *A. oligospora* and other NT fungi.

### 3.4. Ca^2+^-Related Signaling Pathway Regulates Trap Formation

Multifunctional Ca^2+^/calmodulin-dependent protein kinases (CaMKs) are necessary elements in the G-protein signaling pathway. Five CaMKs were identified in *A. oligospora*, the number of traps in the five CaMK-encoding genes deletion strains were significantly lower than that of the WT strain, and the trap formation was delayed in Δ*camkB* mutant [43]. In addition, phospholipase C (PLC), a key enzyme in the inositol phospholipid signaling pathway, hydrolyzes phospholipids to produce inositol 1,4,5-trisphosphate (IP3) and diacylglycerol (DAG) [44]. IP3 causes the release of intracellular calcium ions (Ca^2+^), and DAG triggers protein kinase C activation [45]. Recent research showed that the number of traps in Δ*plc2* mutants was reduced and the partial hyphal loop of the traps was irregular [46]. In addition, the low-affinity calcium uptake system (LACS) also played vital role in trap formation, and the deletion of two genes for the LACS transmembrane protein resulted in a 90% trap reduction and no trap formation, respectively [47].

## 4. Compounds as Signal Molecules to Regulate Trap Formation

There is an evolutionary arms race between predators and prey, and just as prey evolves specific strategies to avoid being hunted, predators also evolve stronger predation strategies, such as lure compounds, to ensure adequate food [48]. Normally, NT fungi are saprophytic, but they will become predators in order to maximize the chance of survival when nutrients are deficient, and their lifestyle transitions accordingly. However, NT fungi are non-motile predators, but nematodes can move at will. Hence, NT fungi evolved an ingenious way, “VOCs”, functioning as mimic pheromone, to lure nematodes. These volatile compounds (VOCs) include dimethyl disulfide, (±)2-methyl-1-butanol, 2,4-dithiapentane, S-methyl thioacetate, and methyl 3-methyl-2-butenoate, and especially, methyl 3-methyl-2-butenoate trigger strong sex- and stage-specific attraction in several *Caenorhabditis* species. Correspondingly, the olfactory neuron AWCs of *C. elegans* sensed the odors emanating from NT fungi and responded the attraction [7,49]. Interesting is that the ascarosides, an evolutionarily highly conserved family of small molecules produced by nematodes, downregulated the expression of polyketide synthase gene (*artA*), which in turn promoted the formation of new traps and then resulted in trapping networks. Therefore, the concentration of ascarosides increased as the nematodes approach, which in turn downregulated arthrosporol and 6-methyl-salicylic acid (6-MSA) formation, further causing the hunting ground to be covered with traps. The ascarosides disappear when the nematodes were completely digested, the contents of arthrosporol and 6-MSA returned to normal levels, and the mycelium switched to saprotrophic growth [50].

Trap formation is a highly energy-consuming process, and to conserve energy, NT fungi have to evolve a more effective strategy to regulate the triggering or closing of trap formation [8]: for example, triggering trap formation after sensing signals of nematodes during infection and terminating trap formation when nematodes were fully digested. Small molecular compounds played an important role in this conversion process, such as ascarosides from nematodes [8], and 6-MSA, oligosporons, arthrobotrisins, and arthrosporols isolated from *A. oligospora* and other NT fungi [50,51,52,53,54,55]. Recently, an increasing number of compounds and associated synthetic genes have been investigated. For instance, the latest research demonstrated that the chemical diversity of metabolites increased notably and exhibited species specificity in the process of changing lifestyle from saprophytic to predatory in NT fungi *A. oligospora*, *A. thaumasia*, and *A. musiformis* [56]. Volatile furanone and pyrone metabolites can help *A. oligospora* capture nematodes in the lifestyle transition [12], and abscisic acid was highly effective at enhancing trap formation of *D. stenobrocha* [57]. In addition, 6-MSA is a chemoattractant that can lure nematodes into the fungal mycelium. The *artA* expression can produce 6-MSA in hyphal tips, and was uncoupled from other enzymes required for the conversion of 6-MSA to arthrosporols; moreover, corresponding deletion strains produced more traps, suggesting a negative role of 6-MSA on trap formation in *D. flagrans* [50]. Furthermore, the gene cluster AOL_s00215 plays a key role in the production of arthrosporols in *A. oligospora*, and the number of traps was increased in deletion mutants of most genes in this gene cluster [10,11,58,59,60]. Arthrobotrisins were downregulated in Δ*ric8*, Δ*ras2*, and Δ*rheb* mutants, indicating that G-proteins and small GTPases were involved in regulating the metabolism of arthrobotrisins [27,29]. Simultaneously, ammonia could function as a signaling molecule in NT fungi to trigger trap formation and kill nematodes, disrupting the gene involved in urea transport and metabolism, resulting in the abolition of urea-induced trap formation in *A. oligospora* [61]. Another study also demonstrated that ammonia can induce trap formation as a signal molecule in NT fungi *A. oligospora*, *A. guizhouensis*, *D. phymatopaga*, *D. cionopaga*, and *D. brochopaga* [62]. Furthermore, PKS−TPS hybrid pathway, for biosynthesis of sesquiterpenyl epoxy-cyclohexenoids, involved in trap formation via ammonia metabolism, deletion of most genes in the PKS−TPS hybrid pathway displayed significantly increase in trap formation [9]. Overall, the discovery of multiple compounds enriches our knowledge of the inducers in trap formation, which participate in trap formation as signaling molecules.

## 5. Multiple Cellular Processes Were Involved in Trap Formation

Trap formation of NT fungi was a sophisticated process and required the coordination and cooperation of diverse cellular processes, such as ubiquitin system [63,64], nitrate assimilation pathway [14], pH-sensing receptor [21], the velvet family proteins [65], scaffold proteins [66], lectins [67], actin [68], the striatin-interacting phosphatase and kinase (STRIPAK) [69], adhesin [70], reactive oxygen species [71], glycerol biosynthesis [72], milRNAs [73], woronin body [15], autophagy [17,18,19,20], and cell-to-cell communication and hyphal fusion [13]. Trap formation was affected to varying degrees by the deletion of genes associated with these cellular processes, such as reduction in number, morphological variation, and time delay in formation (Table 2). Interestingly, the organismic interaction between NT fungi and nematodes was just like a dramatic game of hunt or attack. Effectors played critical roles in regulating host cell physiology to promote virulence, biotrophic growth, or symbiosis [74,75]. In NT fungi, certain secreted proteins can be used to modulate the innate immune system of nematodes or target other intracellular processes. For instance, PEFB, a putative fungal virulence factor, was upregulated during nematodes infection and expressed in *C. elegans*, where it was localized to nuclei [13]. In addition, cell-to-cell communication was required for ring closure, the deletion of *sofT* inhibited the anastomosis of normal vegetative hyphae, resulting in spiral hyphae, while the mutant was still able to trap *C. elegans* [13]. The STRIPAK complex is a highly conserved signaling hub involved in the regulation of hyphal fusion. Deletion of the STRIPAK component SIPC resulted in failure to form complete loops and the formation of column-like trap structures with elongated compartments [69]. On the other hand, nitrogen plays a vital role in the growth of fungi, and autophagy was required for nitrogen homeostasis and recycling [19]. Deletion of *atg8* not only abolished the autophagy induced by nematodes but also suppressed trap formation and reduces pathogenicity of *A. oligospora* [19]. Likewise, the formation of autophagosomes and traps were defective in Δ*atg1*, Δ*atg4*, and Δ*atg5* mutants [17,18,20].

## 6. Summary and Perspectives

### 6.1. Multiple Signaling Pathways and Cellular Processes Co-Regulate Trap Formation

The development of multi-omics contributes to elucidating the mechanism of trap formation. Comparative genomics of nine NT fungi including two *A. oligospora* with different genetic backgrounds revealed commonalities and specificities of trap formation among different NT fungi [5,6,13,22,23,24]. As previously mentioned, the results of comparative genomics and multi-omics consistently showed that signal transduction pathways were closely related to the morphogenetic triggering process of traps. As a representative species of NT fungi, the formation mechanism of traps has been extensively investigated in *A. oligospora*, and trap formation is a complex process, which likely requires the coordination of multiple pathways. This was demonstrated by changes in the expression patterns of relevant genes in various mutant strains and interaction between some proteins (Table 2).

It is the nature of predators to sense and respond to prey. The G-protein signaling pathway was essential for the signal reception and transduction of nematodes in NT fungi. Deletion of *gpb1* resulted in a remarkable reduction in trap formation [24]. Phosphorylation assay showed that phosphorylation signals were lost in both Δ*gpb1* and Δ*fus3* mutants upon nematode induction, suggesting that *gpb1* may function upstream of *fus3* [4]. RGSs negatively regulated G-protein signal transduction, and in the Δ*rgs* mutants, the intracellular cAMP levels were significantly increased and the transcription levels of G-protein signaling genes were downregulated [26]. In addition, RIC8 regulated the cAMP level by interacting with Gα1, thereby participating in the fungal growth, environmental adaptation, and trap formation [27]. These findings show that G-protein regulates the cAMP/PKA and MAPK signaling pathways under the regulation of RGSs and other regulators.

In addition, the small GTPases family acts as switches in the signaling hub of molecular circuits [76]. A recent study showed that the expression levels of genes encoding regulatory subunits of PKA, MAPK, and P21-activated kinases were downregulated in Δ*cdc42* and Δ*rac* mutants; thus, PKA, P21-activated kinases, and HOG1 may be downstream effectors of RHO GTPases in *A. oligospora* [30]. Similarly, RAS GTPases affected MAPK signaling by interacting with STE50 and directly regulating intracellular cAMP levels and the mTOR signaling pathway [29]. These indicate that small GTPases can directly or indirectly regulate MAPK, TOR, and cAMP/PKA signaling pathways. Transcription factors located downstream of these signaling pathways activate or repress the transcription of corresponding genes after receiving cascaded signals, thereby regulating the biological response of cells to external signals. STUA functions downstream of the cAMP/PKA signaling pathway, and the expression of genes involved in the G-protein signaling pathway were transcriptionally repressed in the mutants [42]. Meanwhile, reverse genetics demonstrated that STE12, which regulated the expression of “nematode-responsive genes” that trigger trap formation, was activated by the MAPK cascade in *A. oligospora*. GO enrichment analysis of STE12-dependent differentially expressed genes revealed that the two most significant terms were “integral component of membrane” and “oxidation–reduction process”, which were critical for hyphal growth and virulence in the NT fungi [4]. These results indicated that multiple signaling pathways co-regulate trap formation.

What can be clearly seen in recent studies is the deletion of almost all of these genes affected redox processes [26,27,28,30,34,37,42,71] and partly influence heat shock response [29], cell walls [26,27,28,32,34,41,42], and mitochondria [29,30], indicating the genes related to these processes might be involved in trap formation, which was confirmed in our latest research (unpublished). At the same time, each gene does not influence the trap formation alone: They need to cooperate with each other (Table 2).

**Figure 1:**
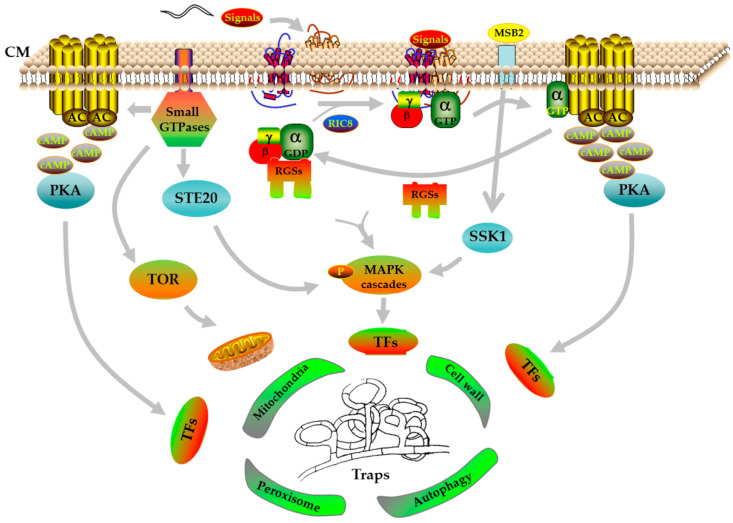
A proposed model for trap formation in NT fungi using *A. oligospora* as an example. CM, cell membrane; α, β, and γ, G-protein subunits; AC, adenylate cyclase; cAMP, cyclic adenosine monophosphate; PKA, protein kinase A; RGSs, regulators of G-protein signaling; RIC8, resistance to inhibitors of cholinesterase; MAPK, mitogen-activated protein kinase; STE20, serine/threonine protein kinase; TOR, mammalian target of rapamycin; MSB2, mucin family signaling protein; SSK1, response regulator; TFs, transcription factors; P, phosphorylation.

Collectively, given the recent progress in reverse genetics, knowledge obtained from different pathways based on the multi-omics and RT-PCR would contribute significantly to elucidating the specific mechanism of the trap formation, which we are only just beginning to understand. In general, it seems that GPCRs are the receptors to respond the nematode’s signals, and the G-protein signaling pathway accurately transform extracellular signals into intracellular signals with the regulation of RGSs, and then transfers to downstream cascade pathways, such as the cAMP/PKA pathway. Simultaneously, the small GTPase family also regulates the MAPK cascades by interacting with STE20 protein kinases. Subsequently, these cascade pathways further regulate a variety of cellular processes by regulating downstream transcription factors, thereby triggering trap formation (Figure 1).

### 6.2. The Regulatory Mechanism of Trap Formation May Vary in NT Fungi

Absolutely, it is not difficult to understand that there are differences in the mechanisms of traps formation due to the differences in the morphologies of traps and predatory ways. Comparative genomics of NT fungi with different types of traps have indicated that many species-specific genes and highly expressed uncharacterized orphan genes have expanded during evolution [5,6,22,23,77]. The whole genome blast analysis and the distribution analysis of orthologous gene showed that the putative pathogen–host interaction genes included 80 specific genes in *A. oligospora*. Furthermore, compared with other pathogenic and non-pathogenic fungi, *A. oligospora* contained 53.64% abundant orphan genes [6]. Another study showed that 20% of the genes in the *M. haptotylum* and *A. oligospora* genomes were shared, but importantly, as many as 16% in each genome genes were unique. Moreover, 15 species-specific genes, unique for the *M. haptotylum*, were found in the cohort of the 10-fold upregulated transcripts during infection [23]. Comparative analysis found that 12 multigene families and 23 gene domains were significantly expanded in the NT fungal genomes. Notably, nine of the expanded multigene families lacked significant matches in public databases, suggesting the existence of a potential novel mechanism underlying trap formation [5]. In addition, the traps of *A. oligospora* and most NT fungi must be induced, but the constricting rings of *D. stenobrocha* can form spontaneously, and its genome was more compact than that of *A. oligospora* with rare repeat-induced point mutation and transposon [22]. Therefore, a more suitable interpretation is that these may be involved in the functional specialization of different NT fungi, which means that the mechanism of trap formation may differ among various NT fungi.

### 6.3. Current Status and Future Prospects

The completion of genome sequencing is a milestone for the study of the mechanism trap formation and interaction between the NT fungi and nematodes. Although the genomes of nine NT fungi, including two *A. oligospora* with different genetic backgrounds, have been sequenced [5,6,13,22,23,24], research on other NT fungi is still in its infancy, except for *A. oligospora* and *D. flagrans*. Differences in trap formation between diverse NT fungi remain an interesting topic for further study. There has been considerable progress in the mechanism of trap formation with the development of multi-omics and molecular biology techniques; however, more knowledge still needs to be accumulated for parsing more comprehensive connections in multiple pathways and organelles during trap formation. According to the phenotypes of these gene mutants, it can be found that mitochondria [29,30], peroxisomes [16], nucleus [17,30,39,46], woronin bodies [15], autophagosomes [17,18,19,20], and other organelles [39,46] have changed in varying degrees. So, here, the question arises: How do they cooperate with each other in trap formation? Are they actually involved in trap formation, or are these changes simply due to defects in growth?

NT fungi are important biocontrol resources, and the traps are the key devices to capture nematodes, which will be formed after receiving external signals. Small molecule compounds act as this signal, such as ascarosides [8], abscisic acid [57], ammonia [61,62], and 6-MSA [50], and arthrosporols [50,51,52,53,54,55] were also involved in regulating trap formation. However, the metabolomic analysis showed that a large number of compounds changed during trap formation and nematode infection [27,29,39,46,56]. So, what these compounds are and how they affect trap formation require further study. Could other compounds from nematodes or the environment also affect trap formation? Moreover, signaling pathways have been identified to be involved in trap formation in multiple NT fungi [4,26,27,28,29,30,32,34,37,38,39,41,43,48,78,79], whereas there is a lack of studies about downstream transcription factors, and further research is needed on the functions of upstream receptors located in the cell wall and membrane.

In summary, increasing knowledge about the regulation mechanism of trap formation of NT fungi has been acquired from the phenotypic traits of various mutants and multi-omics analysis in recent years, whereas elucidating the molecular mechanism of trap formation is still ongoing due to the complexity of trap production by multitudinous NT fungi. Here, we reviewed the latest findings on the trap formation of NT fungi, summarized the shortcomings at present, and discussed the emphases in the future. Our review provides a solid basis for elucidating the mechanisms of trap formation and lifestyle transition of NT fungi, and will help to develop more effective anti-nematode agents by genetic modification.

## Figures and Tables

**Table 1:** Genomic features of different NT fungi.

Trapping Devices	Fungi	Genome Size	GC Content (%)	Number of Genes	Reference
Adhesive network	*A. oligospora* ATCC24927	40.07 Mb	44.45	11,479	[6]
Adhesive network	*A. oligospora* TWF154	39.62 Mb	43.96	12,107	[24]
Adhesive network	*D. flagrans*	36.64 Mb	45.5	9927	[13]
Adhesive knob	*M. haptotylum*	40.40 Mb	45.24	10,959	[23]
Adhesive knob	*D. entomopaga*	38.39 Mb	44.9	11,130	[5]
Constricting ring	*D. stenobrocha*	29.02 Mb	52.5	5597	[22]
Constricting ring	*D. brochopaga*	35.43 Mb	49.42	10,234	[5]
Adhesive column	*D. cionopagum*	43.12 Mb	44.3	11,284	[5]
no trapping device	*D. cylindrospora*	37.71 Mb	46.02	10,785	[5]

**Table 2:** A list of characterized genes contributing significantly to trap formation in NT fungi.

Fungi	Mutated Genes	Annotation	Phenotypic Traits			Reference
			Traps	Conidiation	Mycelial Growth	
*A. oligospora*	*gpb1*	G-protein β subunit	Y	N	N	[24]
*A. oligospora*	*flbA*	Regulator of G-protein signaling Regulator of G-protein signaling Regulator of G-protein signaling Regulator of G-protein signaling Regulator of G-protein signaling Regulator of G-protein signaling Regulator of G-protein signaling	Y	Y	Y	[26]
*A. oligospora*	*rgsA*		Y	N	N	[26]
*A. oligospora*	*rgsB*		Y	Y	Y	[26]
*A. oligospora*	*rgsB2-1*		Y	Y	Y	[26]
*A. oligospora*	*rgsB2-2*		Y	N	N	[26]
*A. oligospora*	*rgsB2-3*		Y	Y	N	[26]
*A. oligospora*	*rgsC*		Y	Y	N	[26]
*A. oligospora*	*gas1*	GAS protein	Y	Y	N	[26]
*A. oligospora*	*ras2*	RAS GTPase	Y	Y	Y	[29]
*A. oligospora*	*ras3*	RAS GTPase	N	N	N	[29]
*A. oligospora*	*rheb*	RAS GTPase	Y	Y	Y	[29]
*A. oligospora*	*rab-7A*	RAB GTPase	Y	Y	Y	[28]
*A. oligospora*	*rab-2*	RAB GTPase	N	Y	N	[28]
*A. oligospora*	*rho2*	RHO GTPase	N	N	N	[30]
*A. oligospora*	*rac*	RHO GTPase	Y	Y	Y	[30]
*A. oligospora*	*cdc42*	RHO GTPase	Y	Y	Y	[30]
*A. oligospora*	*pex1*	Peroxisome biogenesis protein	Y	Y	Y	[16]
*A. oligospora*	*pex6*	Peroxisome biogenesis protein	Y	Y	Y	[16]
*A. oligospora*	*mkk1*	MAPK kinase MKK1	Y	Y	Y	[37]
*A. oligospora*	*ste7*	MAPK kinase STE7	Y	Y	Y	[4]
*A. oligospora*	*fus3*	MAPK FUS3	Y	Y	Y	[4]
*A. oligospora*	*ste12*	Tanscription factor	Y	N	Y	[4]
*A. oligospora*	*slt2*	MAPK SLT2	Y	Y	Y	[34]
*A. oligospora*	*hog1*	MAPK HOG1	Y	Y	N	[38]
*A. oligospora*	*msb2*	Mucin protein	Y	N	Y	[38]
*A. oligospora*	*ime2*	MAPK IME2	Y	Y	Y	[41]
*A. oligospora*	*bck1*	MAPK kinase kinase BCK1	Y	Y	Y	[37]
*A. oligospora*	*ric8*	Resistance to inhibitors of cholinesterase	Y	Y	Y	[27]
*A. oligospora*	*stuA*	Transcription factor	Y	Y	Y	[42]
*A. oligospora*	*glo3*	ARF GTPase activator	Y	Y	Y	[32]
*A. oligospora*	*camk*	Ca^2+^/calmodulin-dependent protein kinases	Y	Y	Y	[43]
*A. oligospora*	*ssk1*	Response regulator	Y	Y	Y	[39]
*A. oligospora* *A. oligospora*	*atg1* *atg13*	Autophagy protein Autophagy protein	Y N	Y N	Y Y	[20] [20]
*A. oligospora*	*atg4*	Autophagy protein	Y	Y	Y	[18]
*A. oligospora*	*atg5*	Autophagy protein	Y	Y	Y	[17]
*A. oligospora*	*atg8*	Autophagy protein	Y	Y	Y	[19]
*A. oligospora*	*hex1*	Woronin body major protein	Y	Y	Y	[15]
*A. oligospora*	*gph1*	Glycogen phosphorylase	Y	Y	Y	[72]
*A. oligospora*	*noxA*	NADPH oxidase	Y	Y	Y	[71]
*A. oligospora*	*niaD*	Nitrate reductase	Y	-	Y	[14]
*A. oligospora*	*niiA*	Nitrite reductase	Y	-	Y	[14]
*A. oligospora*	*nrtB*	Nitrate transporter	Y	-	Y	[14]
*A. oligospora*	*nirA*	nitrogen assimilation transcription factor	Y	-	Y	[14]
*A. oligospora*	*mad1*	Adhesin protein	Y	-	-	[70]
*A. oligospora*	*crn1*	Actin cytoskeleton and actin-associated protein	Y	Y	N	[68]
*A. oligospora*	*palH*	pH sensing receptor	Y	Y	Y	[21]
*A. oligospora*	*fig1*	Low-affinity calcium system member	Y	Y	Y	[47]
*A. oligospora*	*ubr1*	E3 ubiquitin-protein ligase	Y	-	Y	[63]
*A. oligospora*	*vosA*	Developmental regulator	N	Y	N	[65]
*A. oligospora*	*velB*	Developmental regulator	Y	Y	Y	[65]
*A. oligospora*	*g276*	Fucose-specific lectin	Y	N	N	[67]
*A. oligospora*	*g207*	F-box protein	Y	Y	Y	[64]
*A. oligospora*	*AOL_s00215g277*	A putatively cupin-like family gene	Y	Y	N	[10]
*A. oligospora*	*AOL_s00215g278*	Cytochrome P450	Y	Y	Y	[60]
*A. oligospora*	*AOL_s00215g279*	Oxidoreductase	Y	Y	Y	[10]
*A. oligospora*	*AOL_s00215g280*	Cytochrome P450	Y	Y	Y	[58]
*A. oligospora*	*AOL_s00215g281*	Amidohydrolase	Y	N	Y	[11]
*A. oligospora*	*AOL_s00215g282*	Cytochrome P450 oxidoreductase	Y	Y	Y	[11]
*A. oligospora*	*AOL_s00215g283*	6-methylsalicylic acid synthase	Y	-	N	[59]
*A. oligospora*	*AOL_s00079g496*	Polyketide synthase	Y	Y	Y	[12]
*D. flagrans*	*artA*	Polyketide synthase	Y	-	N	[50]
*D. flagrans*	*artB*	Cytochrome P450	Y	-	N	[50]
*D. flagrans*	*artC*	Amidohydrolase	N	-	N	[50]
*D. flagrans*	*artD*	Cytochrome P450	Y	-	N	[50]
*D. flagrans*	*sofT*	Hyphal anastomosis gene	Y	-	Y	[13]
*D. flagrans*	*sipC*	STRIPAK complex component	Y	Y	Y	[69]
*D. dactyloides*	*ste12*	Transcription factor	Y	Y	Y	[40]
*M. haptotylum*	*slt2*	MAPK SLT2	Y	Y	Y	[34]

Y: Affect the corresponding phenotype; N: No effect on corresponding phenotype; -: Not mentioned.

## Data Availability

Not applicable.
